# Eye-of-the-Tiger sign is not Pathognomonic of Pantothenate Kinase-Associated Neurodegeneration in Adult Cases

**DOI:** 10.1002/brb3.8

**Published:** 2011-09

**Authors:** Chaw-Liang Chang, Chih-Ming Lin

**Affiliations:** 1 Department of Pediatrics, Cathay General HospitalHsinchu, Taiwan; 2 Department of Neurology, Cathay General HospitalHsinchu, Taiwan

**Keywords:** Neurodegeneration with brain iron accumulation, pantothenate kinase-associated neurodegeneration, pantothenate kinase 2, eye-of-the-tiger sign, PKAN, NBIA, Hallervorden-Spatz syndrome

## Abstract

An eye-of-the-tiger sign is previously known to have one-to-one correlation with pantothenate kinase-associated neurodegeneration (PKAN). Reviewing the literature on this subject, the correlation between eye-of-the-tiger sign and PKAN seems to show an interesting hypothesis that differs from conventional conclusion. We analyze the published papers in an attempt to reflect this trend and illustrate our points with findings in a 39-year-old man. His brain magnetic resonance imaging study shows typical eye-of-the-tiger sign suggestive of PKAN. Genetic analyses revealed no mutations in pantothenate kinase 2.

An eye-of-the-tiger sign is a specific magnetic resonance imaging (MRI) pattern, a key diagnostic feature of pantothenate kinase associated neurodegeneration (PKAN). It is low-signal intensity rings surrounding the central high-signal intensity regions in the medial aspect of bilateral globus pallidus on T2-weighted MRI (Fig. 1). The surrounding hypointensity of the globus pallidus is due to excess iron accumulation. The central hyperintensity is possibly due to gliosis. PKAN, previously known as Hallervorden-Spatz syndrome, is one of the three extrapyramidal disorders associated with increased amount of brain iron, known as neurodegeneration with brain iron accumulation (NBIA). According to the time of onset, PKAN has been classified as early onset (classic) or late onset (atypical). PKAN is caused by mutation of the pantothenate kinase 2 (PANK2), the major causative gene of NBIA. A one-to-one correlation between an eye-of-the-tiger sign and PKAN was reported by Hayflick et al. (2003). There are few PANK2 negative eye-of-the-tiger sign cases being reported (Hartig et al. 2006; McNeill et al. 2008; Kumar et al. 2006; Strecker et al. 2007; Valentino et al. 2006). Here, we described a case.

**Figure 1:**
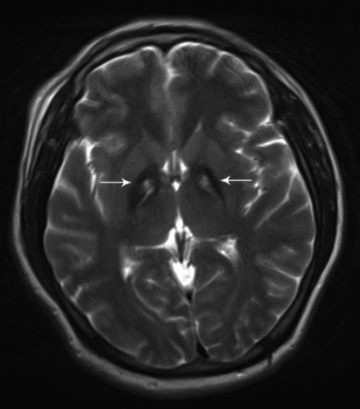
T2-weighted brain MRI of the 39-year-old patient showed bilateral symmetrical hypointensity in the globus pallidus with central hyperintensity, giving an eye-of-the-tiger sign (arrow).

A 39-year-old male patient presented to our hospital with a 3-month history of repetitive, intermittent head turning to right. The symptom showed no fluctuation and did not improve after rest. The physical examination and neurological examination were unremarkable except right cervical dystonia with positive sensory trick. Cervical spine X-ray, nerve conduction velocity, electromyography, electroencephalography, and laboratory evaluation were normal. T2-weighted MRI showed a typical eye-of-the-tiger sign (Fig. 1). This imaging finding suggested PKAN. But, further study of the patient revealed no evidence of PANK2 gene mutation, aceruloplasminaemia, neuroferritinopathy, or retinopathy.

We reviewed published literature and found three major series studying eye-of-the-tiger sign and PANK2 mutation (Table 1) (Hayflick et al. 2003; Hartig et al. 2006; McNeill et al. 2008). And there are several PANK2-negative eye-of-the-tiger sign cases reported (Hartig et al. 2006; McNeill et al. 2008; Kumar et al. 2006; Strecker et al. 2007; Valentino et al. 2006). The correlation between eye-of-the-tiger sign and PKAN was good, 94% (143/152) of eye-of-the-tiger sign had PANK2 mutation, combining the cases of these three series (Hayflick et al. 2003; Hartig et al. 2006; McNeill et al. 2008). All cases with PANK2 mutation have eye-of-the-tiger sign, but not all eye-of-the-tiger sign cases showed PANK2 mutation. One of the PANK2-negative eye-of-the-tiger sign case was multiple system atrophy and two were neuroferritinopathy (Strecker et al. 2007; McNeill et al. 2008).

**Table 1:** PANK2 negative eye-of-the-tiger sign

	Hayflick	Hartig	McNeil	Kumar	Strecker	Valentino P	Our case
Case No./Total	0/69	7/55	2/28	2/2	1/1	1/3	1/1
Age(years)		2,2,4,4,5,10,30	32–69	23,31	65	21	39
Diagnosis		NBIA	neuroferritinopathy		multiple system atrophy	NBIA	NBIA

NBIA, neurodegeneration with brain iron accumulation

We observed most of the PANK2 negative eye-of-the-tiger sign cases were late onset (Hartig et al. 2006; McNeill et al. 2008; Kumar et al. 2006; Strecker et al. 2007) or adult cases (Valentino et al. 2006), early onset PANK2 negative eye-of-the-tiger sign cases were reported only by Hartig et al. (2006). Considering the lower incidence of late onset NBIA (46% in Hayflick et al. 2003 and 25% in Hartig et al. 2006), most of the PANK2-negative cases reported were late onset. The combination of aging process and some other pathologic conditions may lead to an eye-of-the-tiger sign in these adults. We suggest that an eye-of-the-tiger sign might not be interpreted in isolation. Further studies might be necessary before the diagnosis of PKAN, especially in adult cases.
